# Ice slurry ingestion improves physical performance during high-intensity intermittent exercise in a hot environment

**DOI:** 10.1371/journal.pone.0274584

**Published:** 2022-09-15

**Authors:** Akihisa Morito, Takayuki Inami, Akihiro Hirata, Satoshi Yamada, Masatsugu Shimomasuda, Maki Haramoto, Keita Kato, Shigeyuki Tahara, Yuko Oguma, Hiroyuki Ishida, Naohiko Kohtake

**Affiliations:** 1 Graduate School of System Design and Management, Keio University, Kanagawa, Japan; 2 R&D Laboratories, Self-Medication, Taisho Pharmaceutical Co., Ltd., Saitama, Japan; 3 Institute of Physical Education, Keio University, Kanagawa, Japan; 4 Sports Medicine Research Center, Keio University, Kanagawa, Japan; 5 Research & Development Headquarters, Self-Medication, Taisho Pharmaceutical Co., Ltd., Tokyo, Japan; Universidade Federal de Minas Gerais, BRAZIL

## Abstract

Ice slurry ingestion enhances exercise performance by lowering the core body temperature. However, an operational issue related to this ingestion is the requirement for a high intake of 7.5 g·kg^-1^ to produce the desired effects. We investigated the effects of the intake of low amounts of ice slurry at −2°C on the tympanic temperature and exercise performance during repeated high-intensity intermittent exercises in a hot environment. This study was a randomized, crossover study, with a 6-day washout period. Twelve university rugby union players performed two 30-min sessions of high-intensity intermittent exercises separated by a 15-min half-time break on a cycle ergometer in a hot environment (28.8°C ± 0.1°C, 49.5% ± 0.6% relative humidity). The participants ingested 450 g of −2°C-ice slurry (ICE), or a 30°C-beverage (CON) having the same composition as ICE, or 30°C-water (WAT) during the half-time break. The tympanic temperature and skin temperature were measured as the physiological data, and the peak power and mean power as the exercise performance data. The tympanic temperature at the half-time break and beginning of the 2^nd^ session was significantly lower in the ICE group as compared with the CON and WAT groups. The skin temperature at the half-time break was significantly lower in the ICE group as compared with the WAT group. While the peak power and mean power during the 2^nd^ session were significantly greater in the ICE group as compared with the CON and WAT groups. Our findings suggest that even the intake of lower amounts, as compared with those used in previous studies, of low-temperature ice slurry can reduce the body temperature and improve the peak power. These results suggest that intake of low-temperature ice slurry as a strategy for internal body cooling is useful for improving endurance exercise performance in hot environments.

## Introduction

Performing exercises in hot environments can lead to excessive increase of the core body temperature, resulting in heat exhaustion or heat stroke, and potentially, serious central nervous system dysfunction [1, 2]. In general, when the core temperature increases, the body tries to maintain its normal temperature by sweating, increasing blood flow and limiting the capacity for physical activity, in order to protect the central nervous system [1, 3]. Cooling strategies are important for athletes, because high core temperatures can impair exercise performance [4–6]. Several cooling techniques have been attempted to achieve improved exercise performance, including immersion in cold water [7–9], and use of ice jackets and ice packs [8, 10, 11]. Recently, intake of ice slurry has drawn attention as an efficient method to obtain internal cooling without excessive reduction of the muscle temperature in hot environments [12, 13].

Cooling strategies adopted in athletes during sports competitions can be divided into two categories, external cooling and internal cooling [10, 13]. External cooling can be applied to the whole body [14] but also to specific body sites, including the head [15], neck [16, 17], torso [18], thighs [19], and palms [20]. Whole-body cooling by cold water immersion or cooling of the torso using ice jackets are common external cooling techniques [21], however the preparation times are long and dedicated facilities are required. Although cold water immersion has been reported to improve the time to exhaustion [14], wearing ice jackets was reported not to reduce the core temperature during intermittent sprint exercises or even improve the exercise performance [22]. In addition, cold water immersion or cooling of the torso and thighs have been reported to also reduce the muscle temperature [19, 23], which may result in reduced subsequent exercise performance. Therefore, intake of ice slurry, as an internal cooling method, may be a more effective strategy for improving the exercise performance during competitions.

Ice slurry refers to a homogeneous mixture of small ice particles and a carrier liquid. Intake of ice slurry before exercise, as compared with intake of cold fluids, leads to a greater reduction of the core temperature, with larger heat-sink effects [24], thereby improving endurance exercise performance in hot environments [14, 25]. According to previous reports, intake of 7.5 g·kg^-1^ of ice slurry (−1°C) before and during exercise attenuated increase of the core temperature and improved the exercise performance [26, 27]. However, application of this cooling method may be difficult during field sports competitions, due to the need for high amounts of intake. In the aforementioned studies, intake of 7.5–22.5 g·kg^-1^ (e.g., 1.25 g·kg^-1^ 12 times, 7.5 g·kg^-1^ once) of ice slurry before exercise or at every break-time during exercise resulted in a significant reduction of the core temperature by 0.4°C-0.7°C [25, 27]. These intake amounts would be equivalent to 525–1575 g/70 kg body mass, and athletes might find it difficult to consume such large amounts of ice slurry during the half-time break in field sports activities, such as rugby union or soccer matches.

Whilst several adjunctive cooling methods, such as concurrent use of an ice vest [28], have been shown to allow reduction in the intake amounts of ice slurry, their use within the constraints of real-world sporting competitions would still represent an operational issue. Therefore, development of a more practical cooling strategy would be desirable. Intake of ice slurry reduces the core temperature by melting in the gastrointestinal tract as it passes from the oral cavity, via the esophagus, into the stomach. In order to obtain a sufficient cooling effect, it is necessary for a sufficient amount of the ice slurry to reach the stomach without melting. Therefore, we considered that lowering the temperature of the ingested ice slurry might be a more effective cooling strategy than increasing the amount of intake. Tabuchi et al. [29] reported that the use of carbohydrates and electrolytes as solvents may reduce the freezing point of ice slurry and aid in keeping the temperature of the ice slurry below −1°C. Moreover, intake of 5 g·kg^-1^ of low-temperature ice slurry (−1.7°C) prepared using a carbohydrate-electrolyte solution, which is lower than the intake amount prescribed in previous studies, before exercises attenuated the increase in the core temperature in firefighters [29]. However, the possible adverse effects of intake of low-temperature ice slurry (e.g., headache and abdominal pain) have not been reported in previous studies [30, 31].

There are currently no studies on the effects of ice slurry ingestion during the half-time break in rugby union or soccer matches, which involve performance of high-intensity intermittent exercises before and after the half-time break. Adoption of recovery strategies during the short 15-min half-time break is particularly important during, for example, a rugby union match, which is classified as a full-contact sport. Tackles and collision-based activities, which occur during both match play and training in rugby union, have a high energy cost [32] and can result in muscle damage and inflammation [33, 34]. Several studies have investigated the cooling effects of intake of ice slurry to reduce the core temperature and improve endurance exercise performance in hot environments, although the intake of large amounts of ice slurry was needed in these studies [27, 35].

The purpose of the present study was to determine whether intake of low-temperature ice slurry in even smaller amounts than those examined in previous studies during the half-time break might lower the body temperature to improve the high-intensity intermittent exercise performance in athletes. It was hypothesized that lowering the ice slurry temperature would reduce the amount necessary to lower the core temperature sufficiently and improve high-intensity intermittent exercise performance.

## Material and methods

### Participants

Twelve male non-heat-acclimatized healthy university rugby union players (mean ± standard deviation; age: 19.8 ± 1.2 years; age range: 18–22 years; height: 1.72 ± 0.05 m; body mass: 86.3 ± 13.4 kg, body mass index: 29.1 ± 4.2, VO_2_ max: 49.2 ± 3.4 mL·kg^-1^·min^-1^) volunteered to participate in this study, which was conducted between February and March 2020 (Kanagawa, Japan). All the participants provided written informed consent prior to commencement of the study, after receiving a full explanation about the risks and benefits of participating in this study. None of the participants were smokers or had any injuries. This study protocol was approved by the Ethics Committee of Keio University Graduate School of System Design and Management (approval number: SDM-2020-E001) and the Ethics Committee of the Keio University Sports Medicine Research Center (approval number: 2019–06) in Japan, and the study protocol was registered with the University Hospital Medical Information Network Clinical Trials Registry (UMIN000039272). This study conducted in compliance with the latest version of the Declaration of Helsinki.

### Experimental design

This study had an open randomized crossover design, in which the subjects underwent three experimental trials with a 6-day interval between consecutive trials; during each of the trials conducted by the same procedure, the participants ingested either −2°C-ice slurry (ICE), or a 30°C-beverage with the same composition as ICE (CON), or 30°C-water (WAT). The participants completed one familiarization session before the first experimental trial. For each participant, the three experimental trials were implemented at the same time of the day, on the same day of the week to standardize the influence of the circadian rhythm. All trials were conducted between February and March 2020, to limit heat acclimation, in a temperature-controlled room to simulate hot conditions (28.8°C ± 0.1°C, 49.5% ± 0.6% relative humidity), in Kanagawa, Japan. Throughout the study period, the participants maintained their normal lifestyles, including their physical activity and nutritional habits. Participants were instructed to avoid strenuous exercises, alcohol consumption, and intake of any supplements from the day before the start of the study to the end of the study.

### High-intensity intermittent exercise

**Fig 1:**
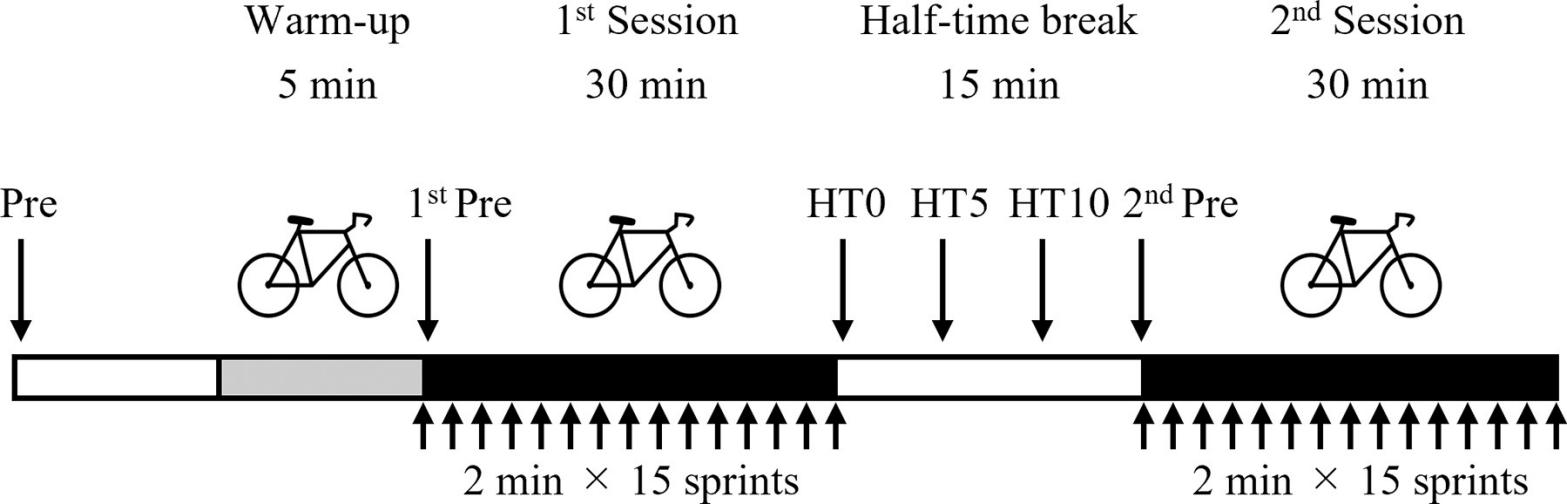
Experimental protocol. HT0: start of the half-time break; HT5: 5 min after HT0; HT10: 10 min after HT0. Each 30-min session consisted of 15 sprints (i.e., maximal pedaling), each lasting 6 sec, interspersed by 114 sec of low work rate pedaling. The two 30-min sessions were separated by a 15-min break at half-time. Participants ingested 150 g of −2°C-ice slurry, or a 30°C-beverage, or 30°C-water every 5 min (total 450 g) during the 15-min half-time break.

Fig 1 shows the experimental protocol. Participants completed a laboratory-based high-intensity intermittent exercise protocol [36] designed to replicate the exercise demands of actual intermittent athletic matches, such as rugby union matches. The initial warm-up consisted of 5 min of pedaling at 100 W and 60 rpm on a Fujin-Raijin cycling ergometer (O.C.Labo, Tokyo, Japan). Participants performed two sessions of 30-min high-intensity intermittent exercises, separated by a 15-min half-time break. The 30-min intermittent exercise sessions consisted of 15 repetitions of a 2-min exercise set. Each 2-min exercise set consisted of 6 sec of maximal pedaling at the load of body mass × 0.075 kp (kilopond), and 114 sec of pedaling at 1.3 kp and 60 rpm (revolutions per minute). The participants drank 100 mL of room-temperature water every 10 min while pedaling.

### Cooling intervention

The participants ingested 450 g of ICE (−2°C), CON (30°C) or WAT (30°C) during the 15-min half-time break, with the amount divided into three equal portions to be ingested every 5 min. The participants ingested the first portion immediately after the end of the 1^st^ session (HT0), the second portion 5 min after HT0 (HT5), and the remaining portion 10 min after HT0 (HT10). The compositions of ICE and CON were the same: carbohydrate 56 kcal·100 g^-1^, Na^+^ 94.0 mg·100 g^-1^, K^+^ 20.3 mg·100 g^-1^.

### Measurements

The body mass (kg) of the participants was measured before and after the exercise sessions using an InBody770 body composition analyzer (InBody Co., Ltd., Seoul, Korea). The tympanic temperature (T_ty_) was continuously recorded using an infrared sensor, CE Thermo probe (Nipro Corp., Osaka, Japan; resolution 0.01°C and accuracy ± 0.1°C) [37]. Tympanic temperature is known to be correlated with the rectal temperature [37] and brain temperature [38, 39], although it has been reported to be lower than the rectal temperature during exercise [40]. Heat storage capacity was calculated using the following formula proposed by Adams et al.: Heat storage capacity (W·m^-2^) = 0.965 (W·kg^-1^°C^-1^) × m × ΔT_ty_·AD^-1^; m: body mass; ΔT_ty_: tympanic temperature change; AD: body surface area [41]. The body surface area was calculated using the following formula proposed by Du Bois et al.: AD = 0.202 × m^0.425^ × height^0.725^ [42]. The skin temperatures in the chest, forearm, and thigh were continuously recorded using Thermocron Type-SL loggers (KN Laboratories Inc., Osaka, Japan; resolution 0.01°C and accuracy ± 0.05°C), which were affixed to the skin using hypoallergenic polyacrylate adhesive tape. The skin temperature (T_sk_) was calculated using the formula proposed by Roberts et al. [43]: T_sk_ = 0.43 × (chest temperature) + 0.25 × (forearm temperature) + 0.32 × (thigh temperature) [44–46]. An optical POLAR OH1 heart rate monitor (Polar Electro, Inc., Lake Success, NY, USA) fixed to the participants’ left upper arm recorded the heart rate every 1 min. The Polar OH1 has been shown to accurately measure the heart rate during moderate to high intensity physical activities, with a mean bias of 0.27 beats·min^-1^ and 95% limits of agreement (upper arm −4.49, 5.15) compared with an electro-cardiographic monitoring device [47]. All devices were affixed to the body at least 30 min prior to the start of the exercises. Subjective fatigue was evaluated using a 10-cm linear visual analogue scale (VAS; 0 [no fatigue] to 10 [total exhaustion]) [48] and Borg’s 6- to 20-point rating of perceived exertion (RPE) before and during the exercise [49]. The mean and peak power output were continuously recorded during each sprint of maximal pedaling at a sampling rate of 10 Hz. The changes of the peak power and mean power during the 2^nd^ session (after the half-time break) were calculated relative to the values at the end of the 15^th^ sprint of the 1^st^ session. The total peak power and total mean power were calculated as the cumulative percent changes of the peak power and mean power, respectively. Adverse events related to the ice slurry ingestion were assessed by self-reported symptoms of the participants during the half-time break and after the exercise.

### Statistical analysis

Descriptive data are presented as means ± standard errors (SE), unless stated otherwise. All statistical analyses were performed using the SAS software package version 9.4 (SAS Institute Japan Ltd., Tokyo, Japan). Normality of the data and homogeneity of variance were tested using Shapiro-Wilk’s test and Bartlett’s test. Differences in the means of the measured values among the beverage conditions and among different time-points were analyzed by Friedman’s two-way ANOVA. When significant differences were found, post-hoc analysis was performed using Wilcoxon’s signed-rank test, with Holm’s adjustment applied for multiple comparisons. P<0.05 was set as the significance level for all the analyses.

## Results

### High-intensity intermittent exercise

**Fig 2:**
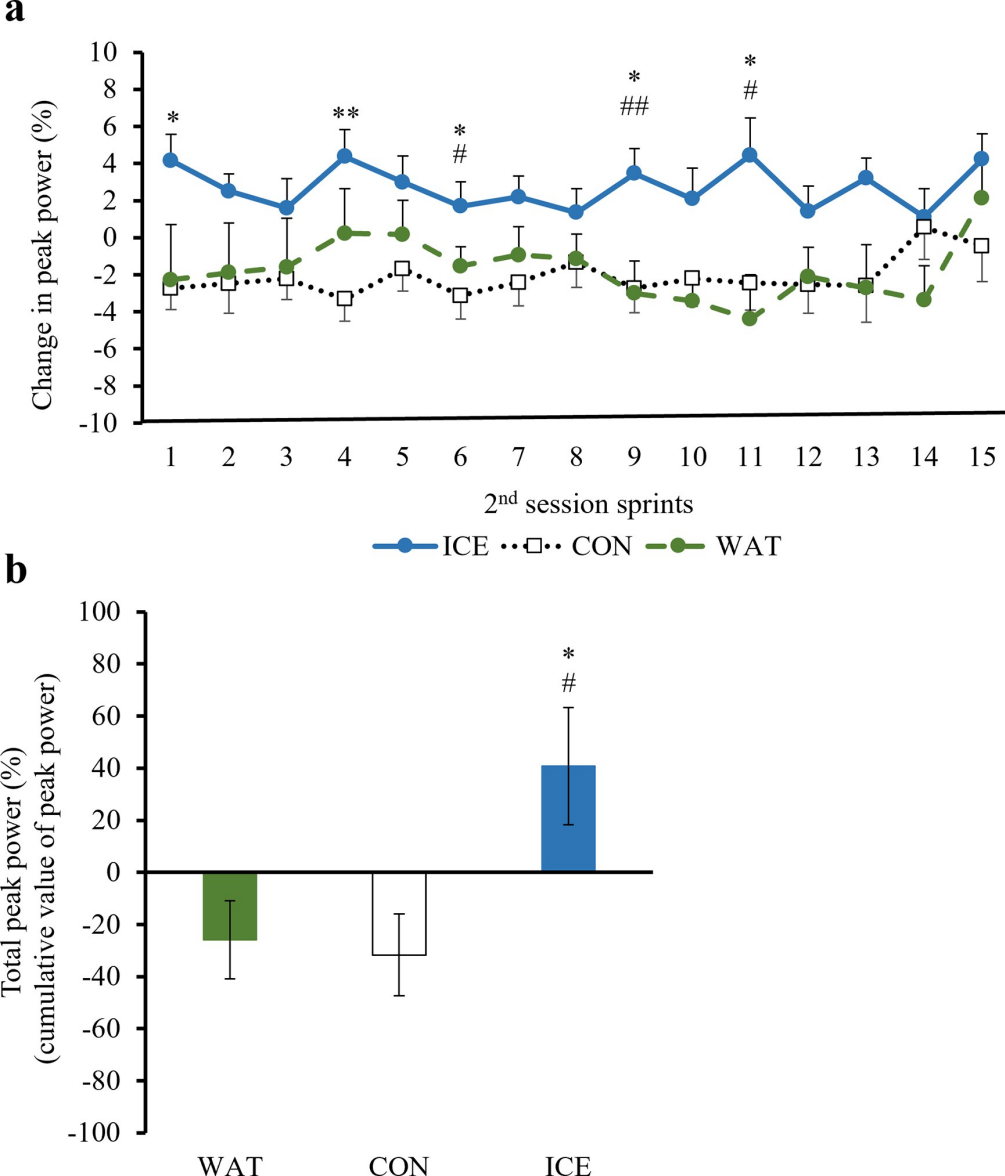
Peak power. ICE: −2°C-ice slurry; CON: 30°C-beverage; WAT: 30°C-water. The changes in the peak power (a) and total peak power (b) during the 2^nd^ session in the ICE, CON, and WAT groups. The changes in the peak power (%) were calculated relative to the value at the end of the 15^th^ sprint of the 1^st^ session. Values are expressed as the means ± SE of the 12 participants, *p < 0.05, **p < 0.01 ICE vs. CON, #p < 0.05, ##p < 0.01 ICE vs. WAT.

**Fig 3:**
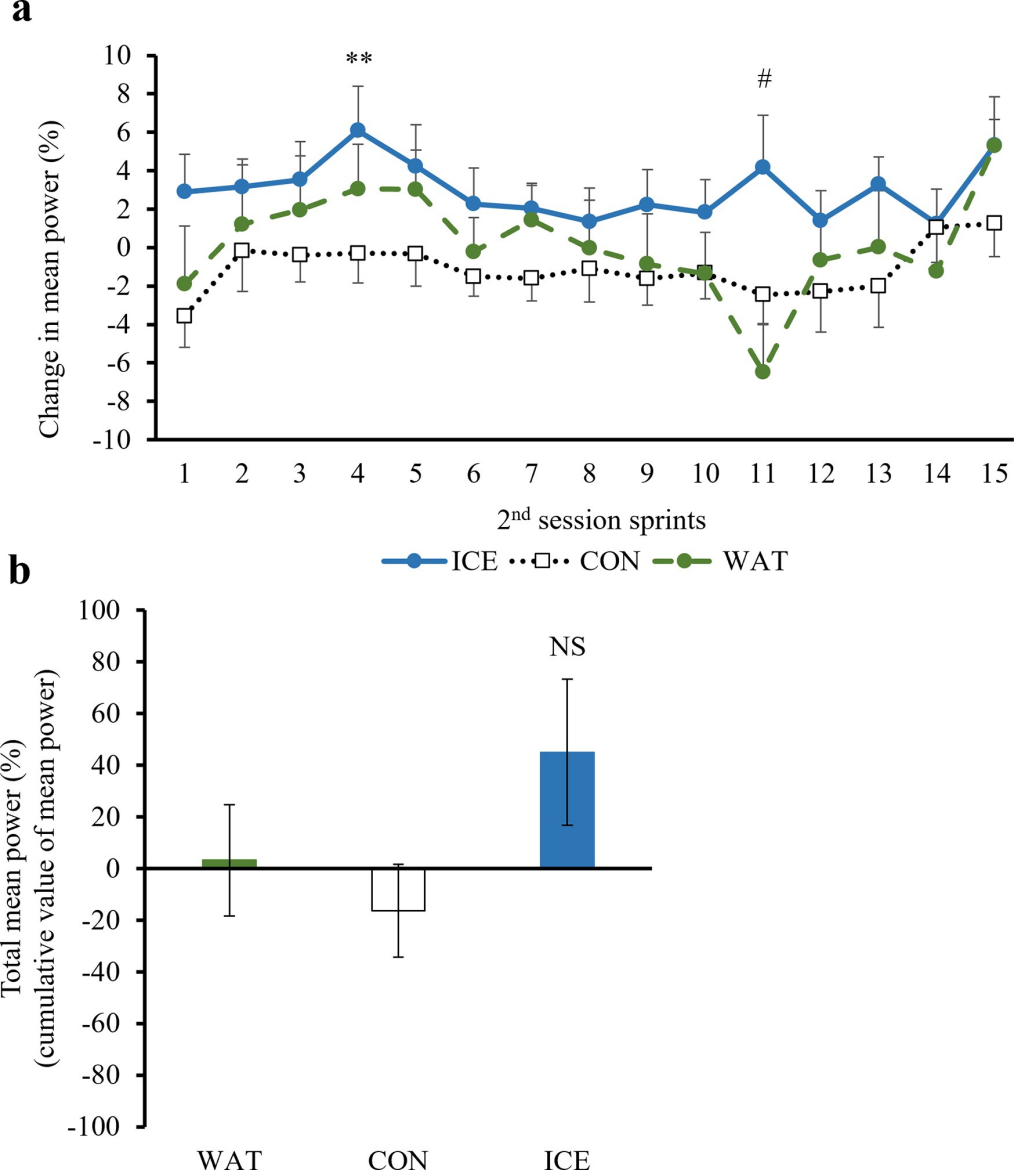
Mean power. ICE: −2°C-ice slurry; CON: 30°C-beverage; WAT: 30°C-water. The changes in the mean power (a) and total mean power (b) during the 2^nd^ session in the ICE, CON, and WAT groups. The changes in the mean power (%) were calculated relative to the value at the end of the 15^th^ sprint of the 1^st^ session. Values are expressed as the means ± SE of the 12 participants, **p < 0.01 ICE vs. CON, #p < 0.05 ICE vs. WAT, NS: Not significant.

During the 1^st^ 30-min session of the exercise protocol, there were no significant differences in the peak power or mean power among the beverage groups. The changes in the peak power and mean power during the 2^nd^ session calculated relative to the value at the end of the 15^th^ sprint of the 1^st^ session are shown in Figs 2A and 3A (the raw data are presented in S1 and S2 Tables). The cumulative values of the peak power (total peak power) and mean power (total mean power) are shown in Figs 2B and 3B (the raw data are presented in S3 and S4 Tables). The changes in the peak power at the 1^st^ (p = 0.016 vs. CON), 4^th^ (p = 0.001 vs. CON), 6^th^ (p = 0.012 vs. CON, p = 0.016 vs. WAT), 9^th^ (p = 0.021 vs. CON, p = 0.002 vs. WAT) and 11^th^ (p = 0.021 vs. CON, p = 0.027 vs. WAT) sprint, and changes in the mean power at the 4^th^ (p = 0.009 vs. CON) and 11^th^ (p = 0.012 vs. WAT) sprint of the 2^nd^ session were significantly higher in the ICE group as compared with the those in the CON or WAT group. The total peak power was also significantly higher in the ICE group than that in the CON (p = 0.023) and WAT (p = 0.027) groups. There were no significant differences in the total mean power among the groups. No significant differences were observed between the CON and WAT groups.

### Temperature

**Fig 4:**
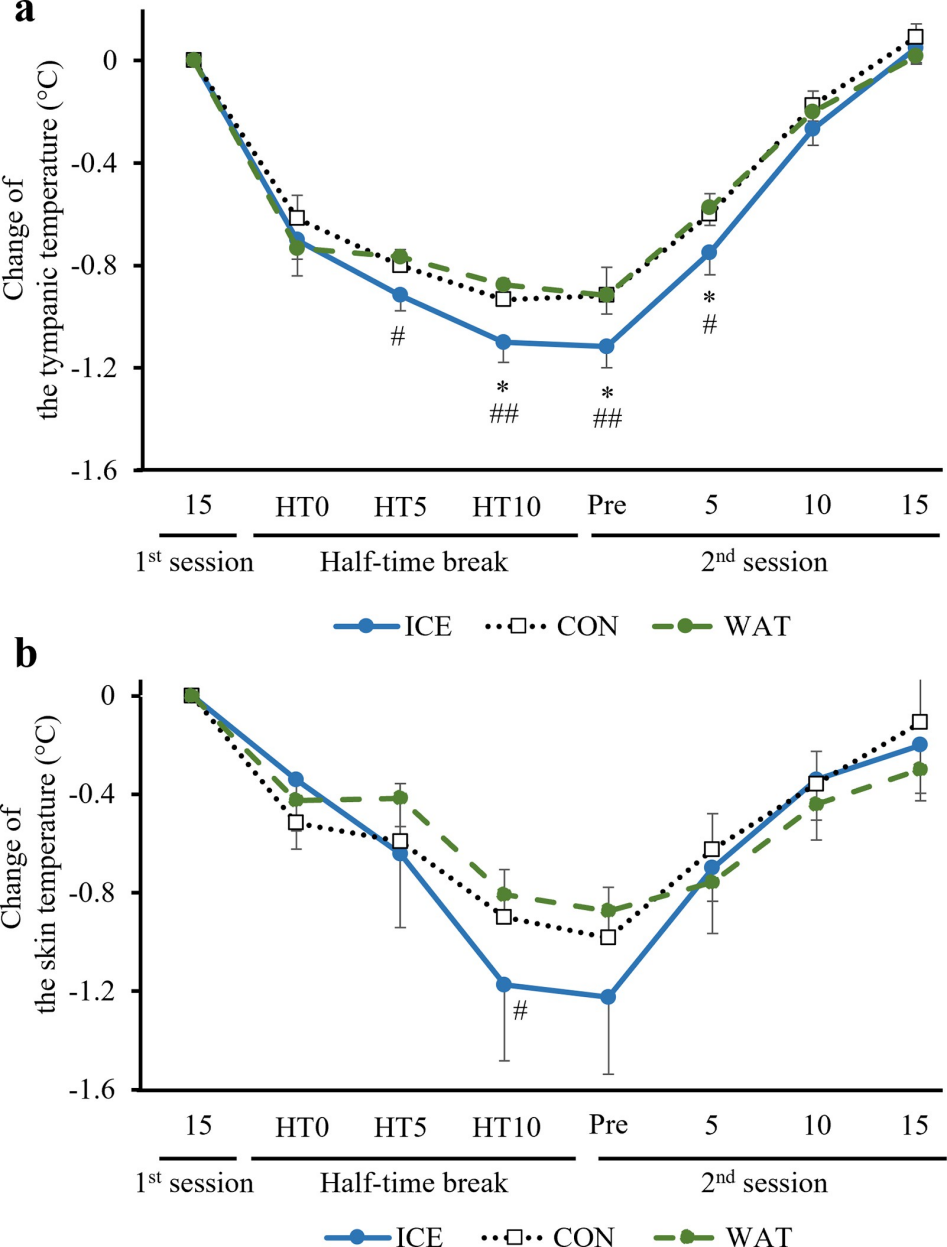
Temperature. ICE: −2°C-ice slurry; CON: 30°C-beverage; WAT: 30°C-water; HT0: start of the half-time break; HT5: 5 min after HT0; HT10: 10 min after HT0. The changes of the T_ty_ (a) and T_sk_ (b) from the 15^th^ sprint of the 1^st^ session to the half-time break and the 2^nd^ session in the ICE, CON, and WAT groups. Values are expressed as the means ± SE of the 12 participants. *p<0.05 ICE vs. CON, #p<0.05, ##p<0.01 ICE vs. WAT.

The changes in the T_ty_ were significantly greater in the ICE group than that in the CON or WAT group at 5 min (p = 0.012 vs. WAT), and 10 min (p = 0.046 vs. CON, p = 0.002 vs. WAT) during the half-time break, Pre 2^nd^ session: (p = 0.030 vs. CON, p = 0.007 vs. WAT), 5^th^ sprint of the 2^nd^ session (p = 0.047 vs. CON, p = 0.017 vs. WAT, Fig 4A; the raw data are presented in S5 Table). The heat storage capacity during the 2^nd^ session (Pre to 15^th^) was significantly higher in the ICE group than that in the CON or WAT group (p = 0.047 vs. CON, p = 0.001 vs. WAT, Table 1). The change in the T_sk_ was significantly greater in the ICE group than that in the WAT group at 10 min (p = 0.04 vs. WAT, Fig 4B; the raw data are presented in S6 Table) during the half-time break. No significant differences were observed between the CON and WAT groups.

**Table 1:** Heat storage capacity.

Variables	Group	2^nd^ session; pre to 15^th^
**Heat storage capacity**	**WAT**	44.2 ± 3.5
	**CON**	44.9 ± 4.0
	**ICE**	50.6 ± 3.4*, ##

ICE: −2°C-ice slurry, CON: 30°C-beverage, WAT: 30°C-water.

Values are expressed as the means ± SE of the 12 participants.

*p < 0.05 ICE vs. CON

##p < 0.01 ICE vs. WAT.

### Heart rate

**Fig 5:**
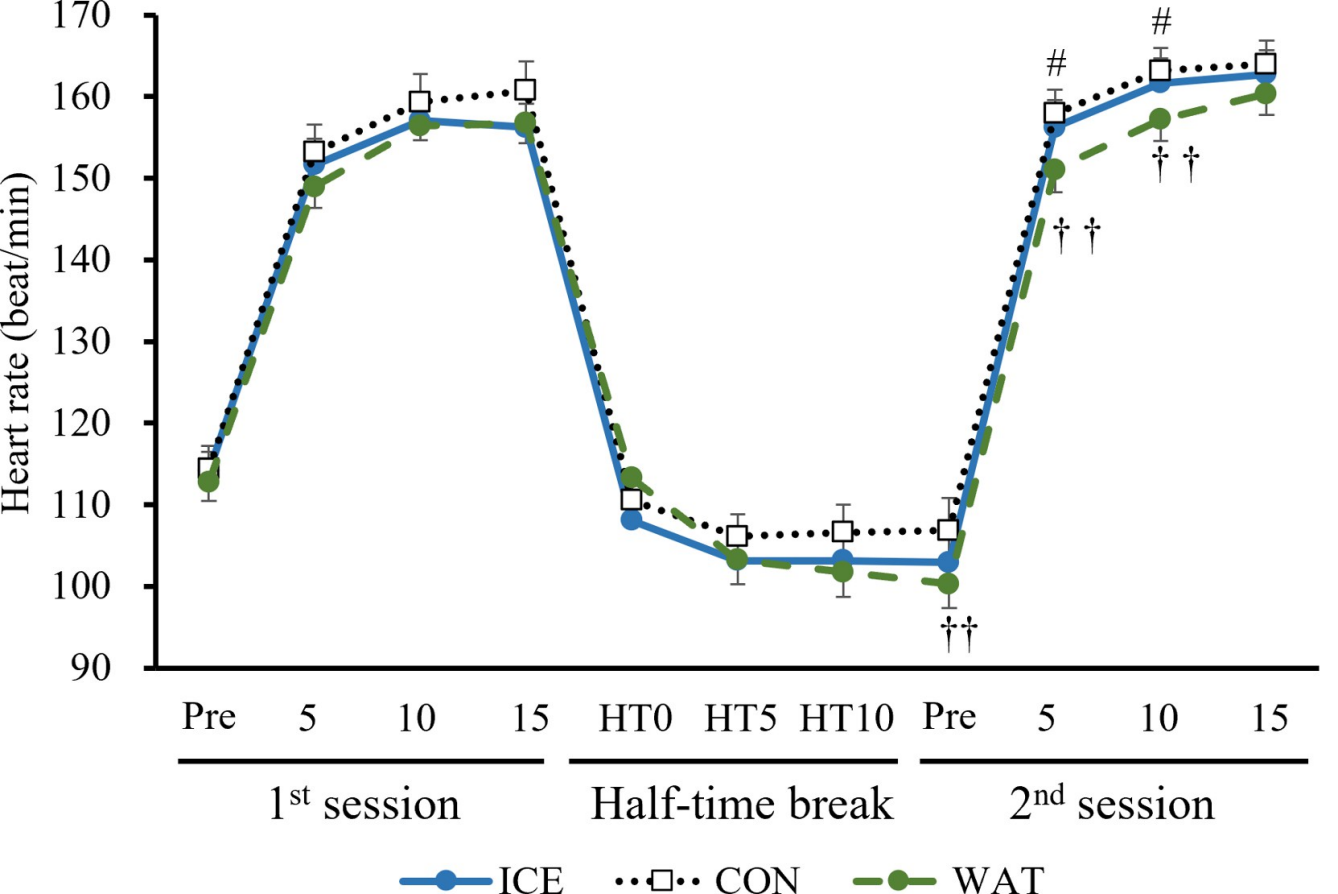
Heart rate. ICE: −2°C-ice slurry; CON:30°C-beverage; WAT: 30°C-water; HT0: start of the half-time break; HT5: 5 min after HT0; HT10: 10 min after HT0. Values are expressed as the means ± SE of the 12 participants. #p < 0.05 ICE vs. WAT, ††p < 0.01 CON vs. WAT.

The heart rate was significantly higher in the ICE or CON group as compared with that in the WAT group at the Pre 2^nd^ session (p = 0.007 CON vs. WAT), 5^th^ (p = 0.013 ICE vs. WAT, p = 0.002 CON vs. WAT) and 10^th^ (p = 0.021 ICE vs. WAT, p = 0.004 CON vs. WAT) sprint of the 2^nd^ session (Fig 5; the raw data are presented in S7 Table).

### Subjective fatigue

There were no significant defferences in the VAS or RPE among the beverage conditions. However, a main effect for time (p < 0.001) was found in the VAS. Likewise, a main effect for time (p < 0.001) was also found in RPE. During the trials, both the subjective scale scores increased significantly over time as compared with Pre, at all time-points (p < 0.05, Table 2).

**Table 2:** Subjective fatigue.

Variables	Group	Pre	1^st^ session sprints			Half-time break	2^nd^ session sprints		
			5^th^	10^th^	15^th^		5^th^	10^th^	15^th^
**VAS**	**WAT**	2.1 ± 0.3	4.5 ± 0.4	6.1 ± 0.4	7.3 ± 0.5	4.3 ± 0.4	6.2 ± 0.5	7.5 ± 0.4	8.2 ± 0.4
	**CON**	2.1 ± 0.3	4.5 ± 0.3	6.1 ± 0.5	7.5 ± 0.5	4.2 ± 0.5	6.4 ± 0.6	7.3 ± 0.5	7.8 ± 0.5
	**ICE**	2.0 ± 0.3	3.9 ± 0.3	6.3 ± 0.5	7.7 ± 0.5	4.7 ± 0.5	6.4 ± 0.5	7.5 ± 0.4	8.3 ± 0.4
**RPE**	**WAT**	9.8 ± 0.4	13.4 ± 0.3	15.5 ± 0.3	17.3 ± 0.3	13.2 ± 0.5	15.3 ± 0.4	17.3 ± 0.3	18.1 ± 0.3
	**CON**	9.6 ± 0.5	13.3 ± 0.4	15.8 ± 0.3	17.5 ± 0.4	12.8 ± 0.5	15.2 ± 0.3	16.9 ± 0.5	17.3 ± 0.6
	**ICE**	9.2 ± 0.5	12.8 ± 0.4	15.7 ± 0.4	17.5 ± 0.4	12.8 ± 0.5	15.3 ± 0.3	17.2 ± 0.3	18.0 ± 0.4

ICE: −2°C-ice slurry; CON: 30°C-beverage; WAT: 30°C-water; VAS: visual analog scale; RPE: rating of perceived exertion.

Values are expressed as the means ± SE of the 12 participants. There were no significant differences among the ICE, CON, and WAT groups. Both subjective scale scores increased significantly over time as compared with Pre, at all time-points (p < 0.05).

### Adverse events

No adverse events (e.g., headache and abdominal pain) related to the ice slurry ingestion were observed during the study period.

## Discussion

This study was conducted in university rugby union players to determine the effects on the body temperature and exercise performance (especially endurance performance) of intake of low-temperature ice slurry. The main findings of this study were that intake of 450 g of low-temperature (−2°C) ice slurry during the half-time break decreased the T_ty_ and improved the exercise performance (total peak power) during the 2^nd^ session, lending support to the hypothesis that even intake of a small amount of low-temperature ice slurry during the half-time break of field sports activity simulating exercises in a hot environment would improve the exercise performance during the 2^nd^ session.

This is the first study to show that intake of low-temperature ice slurry during the half-time break of a rugby union match simulating exercise designed based on the physical demands of repeated-sprint activities in rugby union match play [36, 50], decreases the body temperature and improves the subsequent exercise performance. An operational issue of ice slurry intake was the high intake amount required to obtain a significant effect, as described above. Recently, however, it was suggested that even intake of a small amount of ice slurry prepared using a carbohydrate-electrolyte solution as the carrier may provide a cooling effect [29]. Therefore, in the present study, the participants were asked to ingest a smaller amount of low-temperature ice slurry than that examined in previous studies. If the same assumption as in previous studies were to be used [26, 27], the intake amount of ice slurry needed to alleviate heat stress in the present study would be 647.3 g (7.5 g·kg^-1^), calculated on the basis of the mean body mass of the participants (86.3 kg). However, although the time of intake and exercise protocol differed from that used in previous studies, the change in T_ty_ in the ICE group was significantly greater as compared with that in the other groups even after intake of only 450 g (5 g·kg^-1^) of low-temperature ice slurry [8, 9]. In a previous tennis-simulated study [7], the participants ingested −1°C-ice slurry during every break time (1.25 g·kg^-1^, 12 times) and at the half-time break (7.5 g·kg^-1^), while in the present study, the participants ingested 150 g of −2°C-ice slurry every 5 min during the 15-min half-time break, to avoid adverse events caused by rapid ingestion and to control the temperature of the ice slurry. This intake amount of ice slurry seemed to be effective, because the body temperature decreased to the same degree as that reported in the previous study.

There was a significant recovery effect on the peak power during the second half of the exercise activity, after the half-time break, in the ICE group as compared with that in the CON and WAT groups, while no significant difference was observed between the CON and WAT groups. Therefore, it was considered that the peak power recovery effect may not be due to the intake of carbohydrates in the ice slurry, but to the cooling effect of the low-temperature ice slurry. Increase in the heat storage capacity and decrease in central fatigue due to lowering of the brain temperature has been speculated as the mechanism underlying the effect of the low-temperature ice slurry intake [44, 51]. As with the case in a previous study [27], a significant increase in the heat storage capacity was observed in the ICE group as compared with that in the CON and WAT groups in the present study. Another possible mechanism is the cooling sensation. Trong et al. [52] reported that a cold beverage combined with menthol lessened the performance decline in hot and humid outdoor conditions and that the performances were better with no difference in the psycho-physiological strain (core temperature, heart rate and RPE) between trials. The pleasant stimulus created by ice slurry ingestion may have helped maintain central drive and motivation to exercise and have contributed to improving exercise performance.

Our findings have some potential implications for athletes competing in field sports activities. Intake of low-temperature ice slurry after the 1^st^ session significantly decreased the T_ty_ and T_sk_ as compared with intake of CON or WAT; the T_ty_ and T_sk_ were the lowest at the beginning of the 2^nd^ session (after the intake of low-temperature ice slurry during the 15-min interval) and remained low until the 5^th^ sprint of the 2^nd^ session (25 min after the low-temperature ice slurry intake). Athletes and coaches should consider the lengths of breaks (e.g., half-time break) in each sporting activity to determine the time and amount of ice slurry to be consumed. Since it is difficult to clarify the relationship between the amount of ice slurry ingestion and exercise performance from the results of this study alone, further research is necessary to explore the intake of appropriate amount of ice slurry during break times in each competitive athletic activity.

While discussing the results, some limitations of this study must be recognized. Firstly, we did not measure the core temperature (e.g., esophageal temperature and/or rectal temperature), thermal sensation, or thermal comfort. The cold sensation experienced by the participants in the ice slurry condition could have affected the physical performance [53–55]. Future studies are needed to examine the effects of low-temperature ice slurry ingestion on the core temperature and cold sensation experienced by the athletes to determine the mechanism by which low-temperature ice slurry ingestion improves exercise performance. Secondly, there was no trial of conventional-temperature (−1°C) ice slurry intake. Although a significant difference in performance between the ICE and CON groups was observed in the present study, it was not easy to strictly control and set the 1°C difference while preparing the ice slurry. However, if this can be achieved, it will become possible in the future to further investigate the effects of intake of ice slurry of different temperatures on the performance. Moreover, this study was conducted in an indoor experimental environment with the participants ingesting a pre-determined amount of ice slurry. Even though the high-intensity intermittent exercise used in the present study simulated that in several sports competitions, the physical responses to environmental heat stress may differ between outdoor and indoor environments, because of the radiant heat and convection. In addition, ad libitum ingestion of ice slurry may be more practical in field sports competitions. Further research is needed to clarify whether ad libitum ingestion of low-temperature ice slurry might actually improve the exercise performance in outdoor hot environments.

## Conclusion

In this study, the intake of low amounts of low-temperature ice slurry during the half-time break reduced the T_ty_ and improved the exercise performance in a hot environment. Our data demonstrated that the strategy of internal body cooling via intake of low-temperature ice slurry might be effective for maintaining high exercise performance of athletes in sports competitions, such as rugby union matches, held under severe environmental conditions.

## Supporting information

S1 Table The change in the peak power. (PDF)

S2 Table The change in the mean power. (PDF)

S3 Table Total peak power. (PDF)

S4 Table Total mean power. (PDF)

S5 Table Change of the tympanic temperature. (PDF)

S6 Table Change of the skin temperature. (PDF)

S7 Table Heart rate. (PDF)
