# Supplementary material for: Ice slurry ingestion improves physical performance during high-intensity intermittent exercise in a hot environment
Source: PLoS One. 2022 Sep 15;17(9):e0274584. doi: 10.1371/journal.pone.0274584 (PMC9477354; doi:10.1371/journal.pone.0274584)
Supplement: S1 Table — (PDF) [file pone.0274584.s001.pdf]

**S1 Table. The change in the peak power.**

| | | 2nd session sprints | | | | | | | | | | | | | | |
|---|---|---|---|---|---|---|---|---|---|---|---|---|---|---|---|---|
| | | 1 | 2 | 3 | 4 | 5 | 6 | 7 | 8 | 9 | 10 | 11 | 12 | 13 | 14 | 15 |
| Mean | ICE | 4.15 | 2.51 | 1.60 | 4.37 | 2.99 | 1.69 | 2.20 | 1.36 | 3.46 | 2.09 | 4.44 | 1.43 | 3.20 | 1.12 | 4.23 |
| | CON | -2.73 | -2.48 | -2.22 | -3.31 | -1.68 | -3.13 | -2.42 | -1.34 | -2.73 | -2.20 | -2.45 | -2.54 | -2.59 | 0.56 | -0.45 |
| | WAT | -2.28 | -1.90 | -1.59 | 0.23 | 0.17 | -1.54 | -0.94 | -1.14 | -3.01 | -3.41 | -4.39 | -2.11 | -2.72 | -3.37 | 2.14 |
| Standard error | ICE | 2.98 | 2.68 | 2.65 | 2.41 | 1.84 | 1.05 | 1.53 | 1.34 | 1.74 | 1.38 | 2.40 | 1.61 | 2.35 | 1.86 | 2.23 |
| | CON | 1.16 | 1.60 | 1.12 | 1.19 | 1.19 | 1.26 | 1.26 | 1.33 | 1.32 | 1.51 | 1.46 | 1.55 | 1.99 | 1.74 | 1.92 |
| | WAT | 1.41 | 0.92 | 1.59 | 1.46 | 1.42 | 1.32 | 1.13 | 1.28 | 1.35 | 1.66 | 2.03 | 1.35 | 1.10 | 1.53 | 1.39 |

ICE: -2°C-ice slurry; CON: 30°C-beverage; WAT: 30°C-water.
