# Supplementary material for: Ice slurry ingestion improves physical performance during high-intensity intermittent exercise in a hot environment
Source: PLoS One. 2022 Sep 15;17(9):e0274584. doi: 10.1371/journal.pone.0274584 (PMC9477354; doi:10.1371/journal.pone.0274584)
Supplement: S2 Table — (PDF) [file pone.0274584.s002.pdf]

S2 Table. The change in the mean power.

		2nd session sprints														
		1	2	3	4	5	6	7	8	9	10	11	12	13	14	15
Mean	ICE	2.90	3.15	3.53	6.08	4.23	2.27	2.03	1.35	2.23	1.82	4.16	1.40	3.28	1.24	5.31
	CON	-3.57	-0.16	-0.40	-0.30	-0.33	-1.51	-1.61	-1.10	-1.62	-1.33	-2.46	-2.29	-2.00	1.04	1.25
	WAT	-1.91	1.21	1.94	3.05	3.03	-0.21	1.43	-0.04	-0.86	-1.36	-6.47	-0.66	0.02	-1.25	5.29
Standard error	ICE	3.03	3.10	2.83	2.33	2.05	1.77	1.80	2.50	2.62	2.15	2.46	2.13	2.95	2.71	2.56
	CON	1.63	2.12	1.39	1.54	1.69	1.01	1.16	1.73	1.38	1.34	1.50	2.11	2.15	1.81	1.71
	WAT	1.96	1.46	1.98	2.30	2.17	1.87	1.33	1.76	1.84	1.71	2.73	1.57	1.43	1.79	1.35

ICE: -2°C-ice slurry; CON: 30°C-beverage; WAT: 30°C-water.
