# Supplementary material for: Ice slurry ingestion improves physical performance during high-intensity intermittent exercise in a hot environment
Source: PLoS One. 2022 Sep 15;17(9):e0274584. doi: 10.1371/journal.pone.0274584 (PMC9477354; doi:10.1371/journal.pone.0274584)
Supplement: S3 Table — (PDF) [file pone.0274584.s003.pdf]

S3 Table. Total peak power.

		Total peak power
Mean	WAT	-25.87
	CON	-31.71
	ICE	40.83
Standard error	WAT	14.98
	CON	15.70
	ICE	22.51

ICE: -2°C-ice slurry; CON: 30°C-beverage; WAT: 30°C-water.
