# Supplementary material for: Ice slurry ingestion improves physical performance during high-intensity intermittent exercise in a hot environment
Source: PLoS One. 2022 Sep 15;17(9):e0274584. doi: 10.1371/journal.pone.0274584 (PMC9477354; doi:10.1371/journal.pone.0274584)
Supplement: S4 Table — (PDF) [file pone.0274584.s004.pdf]

S4 Table. Total mean power.

		Total mean power
Mean	WAT	3.21
	CON	-16.38
	ICE	44.98
Standard error	WAT	21.56
	CON	17.96
	ICE	28.31

ICE: -2°C-ice slurry; CON: 30°C-beverage; WAT: 30°C-water.
