# Supplementary material for: Ice slurry ingestion improves physical performance during high-intensity intermittent exercise in a hot environment
Source: PLoS One. 2022 Sep 15;17(9):e0274584. doi: 10.1371/journal.pone.0274584 (PMC9477354; doi:10.1371/journal.pone.0274584)
Supplement: S5 Table — (PDF) [file pone.0274584.s005.pdf]

S5 Table. Change of the tympanic temperature.

		1st session	Half-time break			2nd session			
		15	HT0	HT5	HT10	Pre	5	10	15
Mean	ICE	0	-0.70	-0.92	-1.10	-1.12	-0.75	-0.27	0.05
	CON	0	-0.62	-0.80	-0.93	-0.92	-0.60	-0.18	0.09
	WAT	0	-0.73	-0.77	-0.88	-0.92	-0.58	-0.20	0.02
Standard error	ICE	0	0.08	0.06	0.08	0.08	0.09	0.06	0.06
	CON	0	0.09	0.06	0.08	0.11	0.08	0.06	0.05
	WAT	0	0.11	0.04	0.06	0.07	0.07	0.04	0.03

ICE: -2°C-ice slurry; CON: 30°C-beverage; WAT: 30°C-water; HT0: start of the half-time break; HT5: 5 min after HT0; HT10: 10 min after HT0.
