# Supplementary material for: Ice slurry ingestion improves physical performance during high-intensity intermittent exercise in a hot environment
Source: PLoS One. 2022 Sep 15;17(9):e0274584. doi: 10.1371/journal.pone.0274584 (PMC9477354; doi:10.1371/journal.pone.0274584)
Supplement: S6 Table — (PDF) [file pone.0274584.s006.pdf]

S6 Table. Change of the skin temperature.

		1st session	Half-time break			2nd session			
		15	HT0	HT5	HT10	Pre	5	10	15
Mean	ICE	0	-0.34	-0.64	-1.18	-1.23	-0.70	-0.34	-0.20
	CON	0	-0.52	-0.59	-0.90	-0.98	-0.63	-0.36	-0.11
	WAT	0	-0.43	-0.42	-0.81	-0.87	-0.76	-0.44	-0.30
Standard error	ICE	0	0.28	0.30	0.31	0.31	0.27	0.25	0.23
	CON	0	0.19	0.23	0.20	0.21	0.15	0.13	0.18
	WAT	0	0.12	0.11	0.08	0.09	0.08	0.06	0.10

ICE: -2°C-ice slurry; CON: 30°C-beverage; WAT: 30°C-water; HT0: start of the half-time break; HT5: 5 min after HT0; HT10: 10 min after HT0.
