# Supplementary material for: Ice slurry ingestion improves physical performance during high-intensity intermittent exercise in a hot environment
Source: PLoS One. 2022 Sep 15;17(9):e0274584. doi: 10.1371/journal.pone.0274584 (PMC9477354; doi:10.1371/journal.pone.0274584)
Supplement: S7 Table — (PDF) [file pone.0274584.s007.pdf]

S7 Table. Heart rate.

		1st session				Half-time break			2nd session			
		Pre	5	10	15	HT0	HT5	HT10	Pre	5	10	15
Mean	ICE	114.1	151.6	157.1	156.2	108.1	103.2	103.1	102.9	156.2	161.7	162.7
	CON	114.4	153.2	159.3	160.7	110.5	106.1	106.6	106.8	157.9	163.2	164.0
	WAT	112.7	148.9	156.4	156.7	113.3	103.2	101.7	100.3	151.0	157.2	160.3
Standard error	ICE	2.4	3.2	2.5	2.9	3.2	3.1	3.7	3.6	3.3	3.0	2.9
	CON	2.9	3.4	3.5	3.6	3.2	2.7	3.4	4.0	3.0	2.8	2.9
	WAT	2.2	2.6	1.8	2.5	3.8	3.0	3.0	2.9	2.7	2.6	2.6

ICE: -2°C-ice slurry; CON: 30°C-beverage; WAT: 30°C-water; HT0: start of the half-time break; HT5: 5 min after HT0; HT10: 10 min after HT0.
